# Interdisciplinary research on periodontitis and depression: a bibliometric analysis of research trends, hotspots and future directions

**DOI:** 10.3389/froh.2025.1588737

**Published:** 2025-06-27

**Authors:** Qian Tang, Weiye Xu, Fushen Zhang, Guangyi Yuan, Dian Zhou

**Affiliations:** ^1^ Key Laboratory of Oral Health Research, Xiangya School of Stomatology, Central South University, Changsha, China; ^2^ Department of Anatomy and Neurobiology, School of Basic Medical Sciences, Central South University, Changsha, China; ^3^ Department of Stomatology, Lecong Hospital of Shunde Dental Centre, Foshan, Guangdong, China

**Keywords:** periodontitis, depression, bibliometrics, VOSviewer, CiteSpace

## Abstract

**Background:**

Periodontitis, a chronic inflammatory disease affecting nearly 50% of the global population, has been increasingly linked to depression, a prevalent psychiatric disorder.

**Methods:**

This study conducted a comprehensive bibliometric analysis to explore the association between periodontitis and depression, from 2000 to 2024 via the Web of Science Core Collection (WoSCC) database. Bibliometric parameters were extracted and bibliometric analysis was conducted via VOSviewer, and CiteSpace software.

**Results:**

A total of 205 publications, comprising 173 original articles and 32 reviews, were analyzed via VOSviewer and CiteSpace, with a focus on countries, institutions, authors, journals, keywords, and citations. The results revealed a significant increase in publications, with notable contributions from China, the USA, and Brazil, accounting for 43.9% of all studies. The collaborative networks highlight the growing interdisciplinary nature of this field. “Depression” (*n* = 71), “disease” (*n* = 66), “association” (*n* = 50), “oral health” (*n* = 47) and “stress” (*n* = 37) were the most frequent keywords, reflecting current research hotspots. Through the time map analysis of keyword clustering, we found that the research hotspots gradually changed from “risk factors”, “chronic periodontitis” and “psychosocial factors” to “inflammation”, “Alzheimer's disease” and “smoking” and other keywords. Keyword analyses identify emerging research hotspots, including the interplay of stress, anxiety, and inflammation.

**Conclusion:**

The number of related studies on periodontitis and depression continues to increase. The analysis of countries, authors and keywords reveals development trends, collaboration opportunities, and priority themes such as psychosocial factors and systemic inflammation. These findings provide a theoretical foundation for future research on periodontitis and depression.

## Introduction

Periodontitis is a chronic disease caused by polymicrobial infections in the oral cavity. It is the sixth most common human disease globally, affecting nearly 50% of the world's population, and is the most prevalent inflammatory disease among adults (1, 2). Periodontitis typically manifests as gingival inflammation, ultimately leading to tooth loss and occlusal dysfunction (3). Periodontitis is not only a localized oral inflammatory disease but can also induce low-grade systemic inflammation through the release of pro-inflammatory cytokines and the invasion of periodontal pathogens (4). There is evidence that inflammation is closely related to depression (5), and an increasing number of studies suggest that chronic periodontitis may be a potential risk factor for psychiatric disorders such as depression, anxiety (6–9). Major depressive disorder is a complex disorder with biological, psychological, and social components, and the phrasing should highlight its comprehensive biopsychosocial basis (10, 11). Several cross-sectional studies have found a positive correlation between periodontitis and depression (12, 13). However, the connection and underlying mechanisms between these two diseases remain unknown.

This intersection heralds a promising era of interdisciplinary research linking oral medicine and psychiatry, as oral health may reflect the health of the mental system. Treating oral diseases opens new avenues for curing depression (14–17). Therefore, exploring the complexity between these two fields in greater depth is crucial for comprehensively analyzing related research trends.

This study primarily focuses on evaluating the relationship between periodontitis and depression. Currently, there is no bibliometric study that systematically summarizes and compares the research status and trends of periodontitis and depression. Bibliometrics is a scientific method based on systematic analysis of literature and bibliometric characteristics. CiteSpace and VOSviewer software can be used to evaluate the research status of scientific publications and predict future research trends (18, 19). The Web of Science is the most commonly used database for bibliometric studies, containing more scientific publications than other databases, making it a comprehensive data source for bibliometric analysis (20, 21). Therefore, this review uses CiteSpace and VOSviewer to analyze annual publication volume, countries, institutions, authors, journals, disciplines, and keywords in the literature included in the core WoS database published between 2000 and 2024. Based on the results of bibliometric analysis, research hotspots and development trends in studies on Periodontitis patients with depression were identified. This provides guidance for improving the quality of life and treatment of Periodontitis and depression patients in clinical practice.

## Materials and methods

### Data collection and extraction

**Figure 1:**
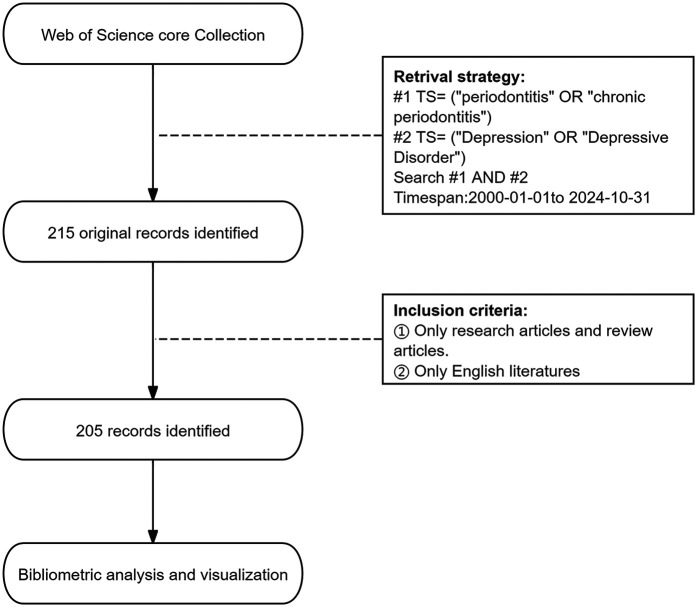
The flowchart.

The literature retrieval process is shown in Figure 1. We conducted an advanced search of the WoSCC database in November 2024, and used the following search terms to identify publications primarily concerning saliva: Topic Search (TS) = (“periodontitis” or “chronic periodontitis”) AND TS = (“Depression” OR “Depressive Disorder”). We limited the search period to between 1 January 2000 and 31 October 2024. The document type was limited to original and review articles, and the publication language was limited to English. The exclusion criteria were as follows. The types of publications are meeting abstracts, editorial materials, corrections, book chapters, letters, and non-English articles. F.Z. and W.X. were responsible for literature retrieval and screening, respectively. In case of disagreements, a third researcher (Q.T.) made the final decision. The identified publications that met the inclusion criteria were exported as plain text files in the format of “Full Record and Cited References”.

### Bibliometric analysis and visualization

VOSviewer (version 1.6.17) (18) was employed to perform co-authorship analyses for countries/regions, institutions, and authors, as well as co-citation analyses for journals, authors, and cited references. Combined with Scimago Graphica, VOSviewer was also applied to analyze and visualize interrelationships among countries/regions. In the network maps generated by VOSviewer, node size represents the quantity or frequency of elements, and links between nodes indicate levels of collaboration or co-citation. Clusters are represented by nodes of the same color, while overlay visualizations demonstrate the temporal progression of node relationships. Purple nodes correspond to earlier publications, whereas yellow nodes indicate more recent works.

We used CiteSpace (version 6.1.R6) (22) to carry out dual-map overlay analysis of journals, keyword co-occurrence, keyword clustering, reference co-citation and clustering analysis, as well as for detecting citation bursts in references and keywords. The co-citation clustering analysis was performed with CiteSpace parameters set as follows: time slices (1999–2023), years per slice (1), and selection criteria (*k* = 25).

## Results

### Analysis of publication and citation trends

**Figure 2:**
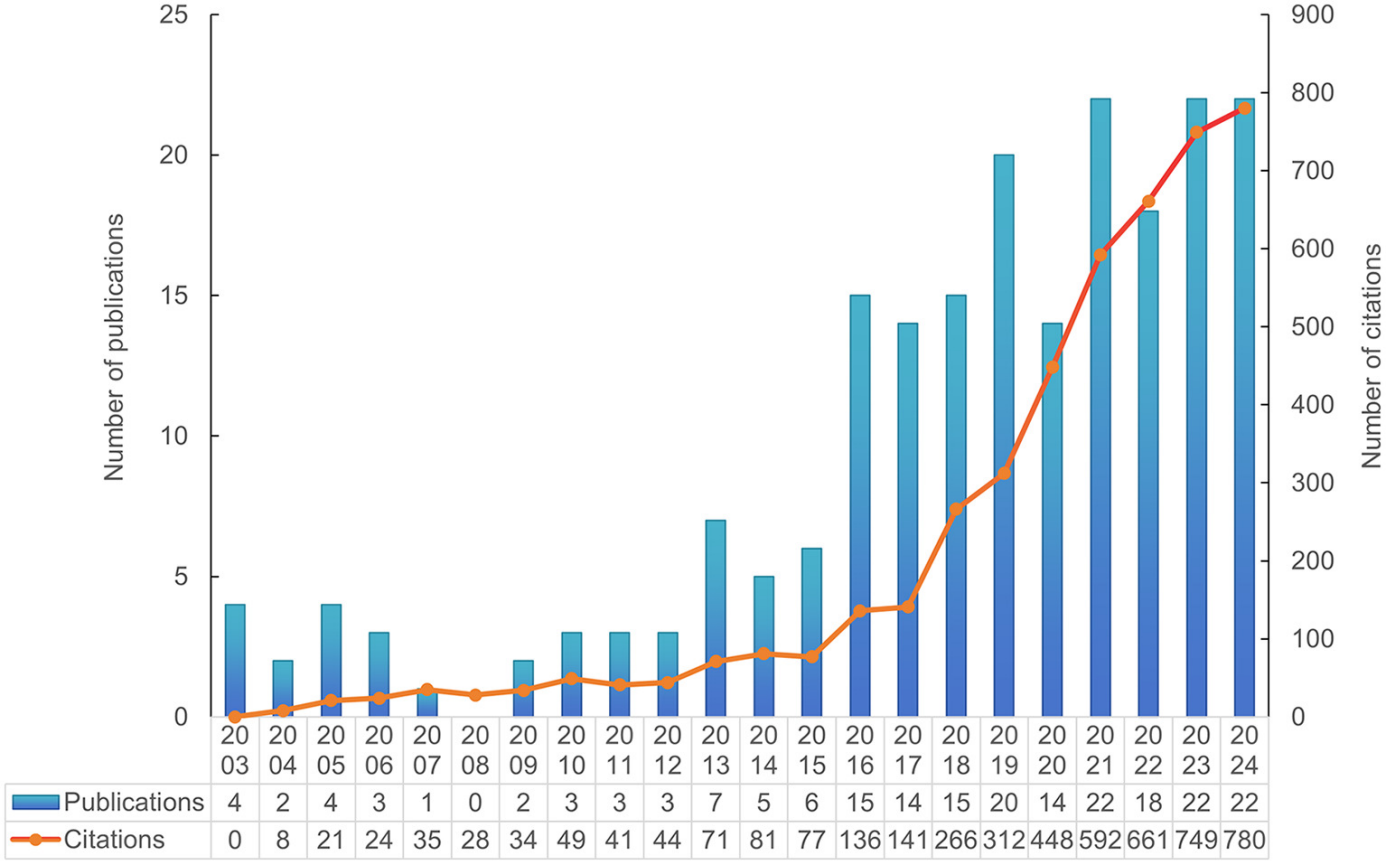
The trend of annual publications and annual citations.

A total of 205 documents published between January 1, 2000, and October 31, 2024 (inclusive) were retrieved from the WoSCC database. The document types were limited to English original research articles and review articles. According to the reference database, 173 research articles and 32 review articles were included. The total number of citations was 4,624, with an average of 22.34 citations per article. The annual publication and citation trends are shown in Figure 2. On the basis of the publication years, the retrieved articles can be divided into two periods: the first period, from 2003 to 2015, saw very few publications per year; the second, an emerging period from 2016 to 2024, had more than 10 publications annually. The year 2024 marked the peak in publication numbers, with 24 articles published. Over the past five years, a total of 98 articles were published, accounting for 47.8% of all the articles. Furthermore, bibliometric parameters including 53 countries or regions, 473 organizations, 111 journals, 2,670 co-cited journals, 1,241 authors, 7,656 co-cited authors, and 1,113 keywords, were carefully analyzed and identified. Among countries, authors from the People's Republic of China, the USA, and Brazil ranked in the top three in terms of the number of publications, with these countries also experiencing rapid growth in publication output.

### Analysis of countries or regions

**Figure 3:**
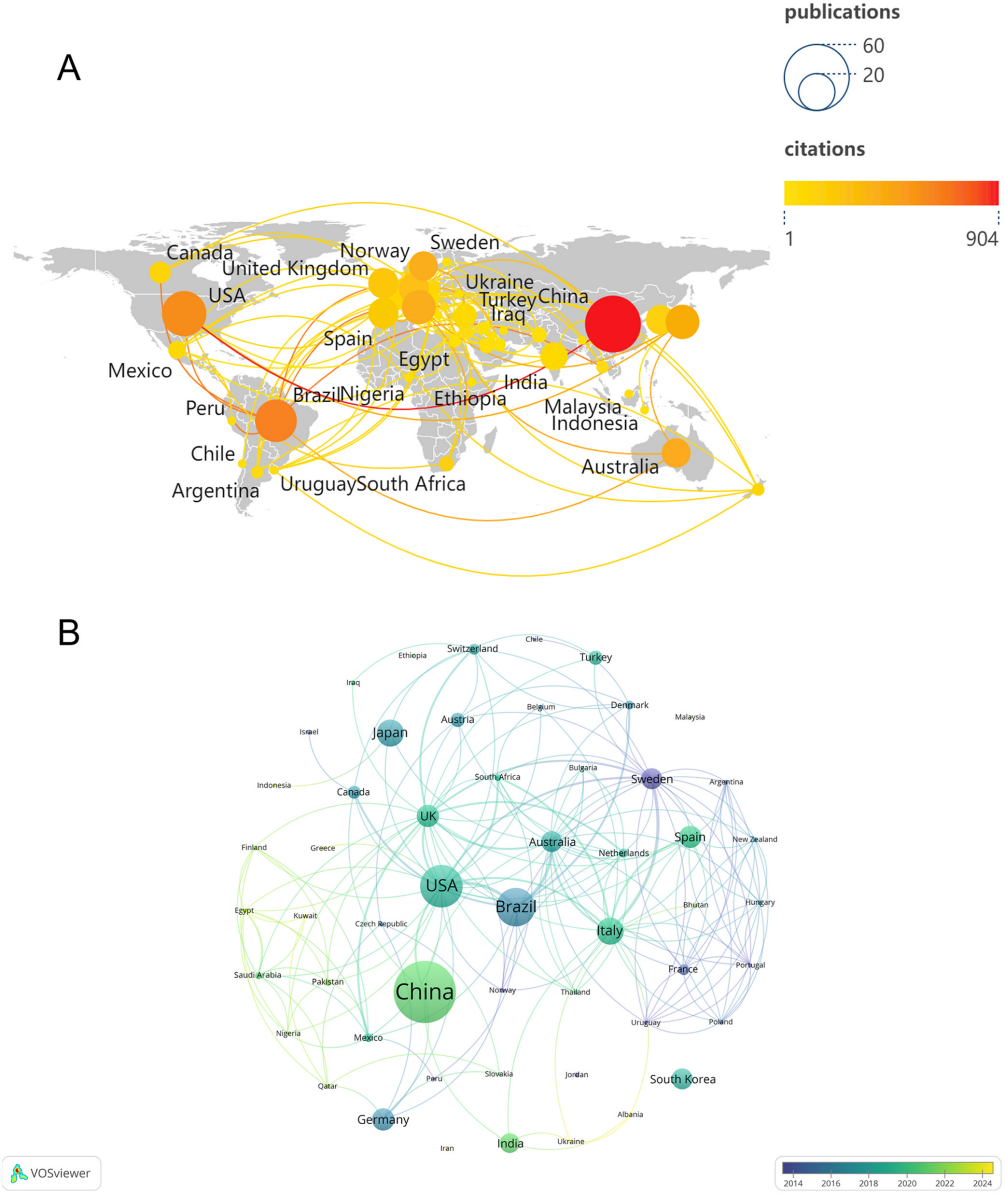
**(A)** global publication and cooperation landscape. **(B)** The co-authorship network timeline of countries.

Over the past two decades, 53 countries or regions have contributed at least one publication in this research field. Figure 3A illustrates the global publication and collaboration patterns, where the node size represents the number of publications, the color indicates the number of citations, and the links between nodes represent collaboration between countries. A total of 48 countries established collaborative relationships, with the USA being the most active in international collaboration in the field of periodontitis and depression, forming partnerships with 23 other countries. Figure 3B displays the time-based network of country co-authorships, with yellow nodes representing countries that have been active in recent years, such as Ukraine and Albania. Additionally, Table 1 summarizes the number of publications, total citations, and average citations for the top 10 countries or regions. Over the past decade, China has become the leading country in terms of publications on periodontitis and depression, with 41 articles published and 904 citations (22.0 citations per article on average), accounting for 20.0% of all papers included in the reference database. The USA published 26 articles with 587 citations (22.6 citations per article on average), followed by Brazil, which published 23 articles with 634 citations (27.6 citations per article on average).

**Table 1:** The top 10 highly documents countries/regions for periodontitis and depression research.

Label	Publications	Total citations	Average citations	Link	Total link strength
China	41	904	22.0	5	6
USA	26	587	22.6	23	30
Brazil	23	634	27.6	10	16
Italy	15	399	26.6	19	25
Japan	15	414	27.6	4	4
Germany	12	255	21.3	6	6
Spain	12	203	16.9	12	13
United Kingdom	12	222	18.5	22	28
Australia	11	402	36.5	20	24
Sweden	11	393	35.7	19	23

### Analysis of institutions

**Figure 4:**
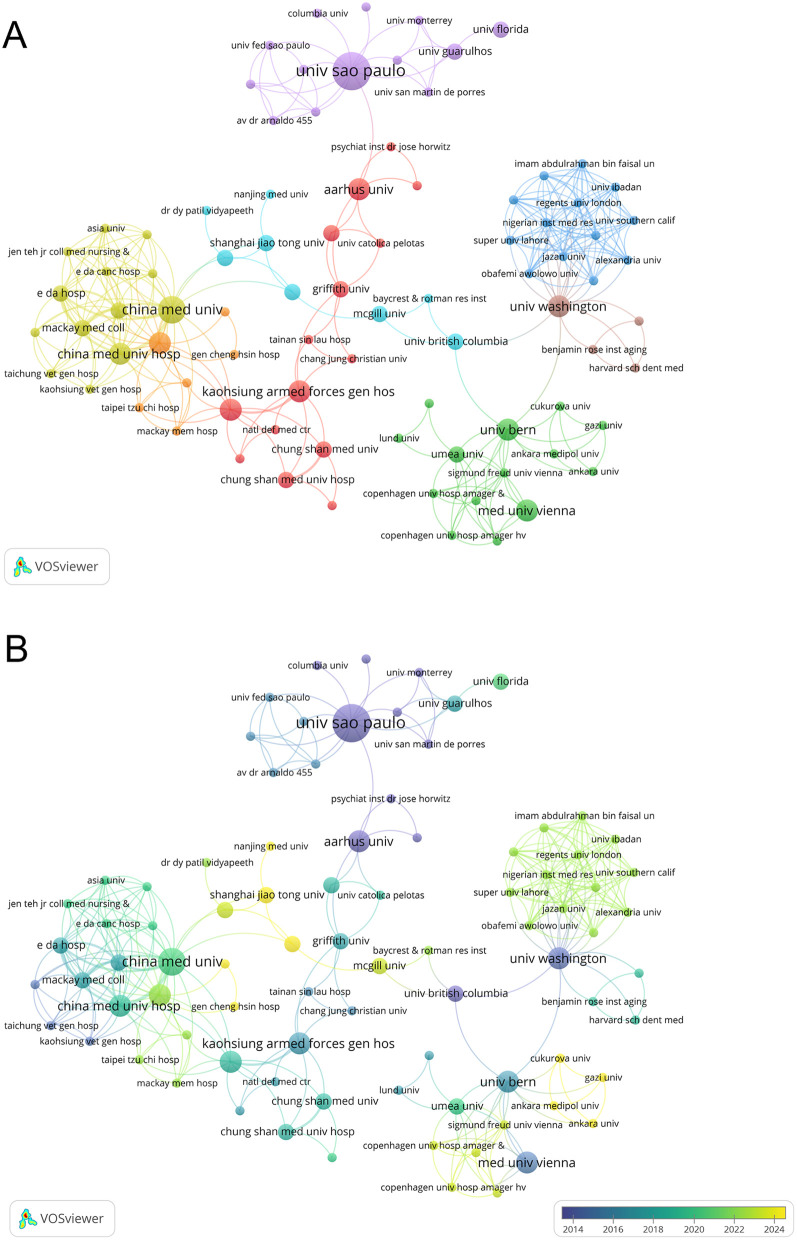
**(A)** The co-authorship network clusters of institutions. **(B)** The co-authorship network timeline of institutions.

A total of 473 institutions contributed to this research field in the form of published articles or reviews. Among them, 10 institutions published more than four papers in the field of periodontitis and depression. Table 2 lists the top 10 research institutions by publication count, accounting for 22.43% of the collected data. The Universidade de São Paulo and the Institute of Health Carlos III are the most significant contributors, each publishing six articles with total citations of 184 and 103, respectively. As shown in Figure 4A, 86 organizations are interconnected, forming a widespread collaboration network. Among them, China Medical University established collaborative relationships with 20 institutions and had the highest total link strength (TLS) of 26. In recent years, Shanghai Jiao Tong University and Nanjing Medical University have made notable contributions to research in depression and periodontitis (Figure 4B).

**Table 2:** Top 10 institutions with the most productive regarding periodontitis and depression research.

Label	Publications	Total citations	Average citations	Links	Total link strength
Inst Hlth Carlos Iii	6	101	16.8	22	28
Univ Sao Paulo	6	184	30.7	12	12
Karolinska Inst	5	182	36.4	3	3
Univ Fed Minas Gerais	5	120	24.0	8	9
China Med Univ	4	40	10.0	20	26
Univ Complutense Madrid	4	78	19.5	24	26
Univ Fed Rio Grande Do Sul	4	74	18.5	7	8
Univ Maryland	4	68	17.0	22	24
Univ Michigan	4	125	31.3	2	2
Univ Queensland	4	200	50.0	15	15

### Analysis of authors and co-cited authorships

**Figure 5:**
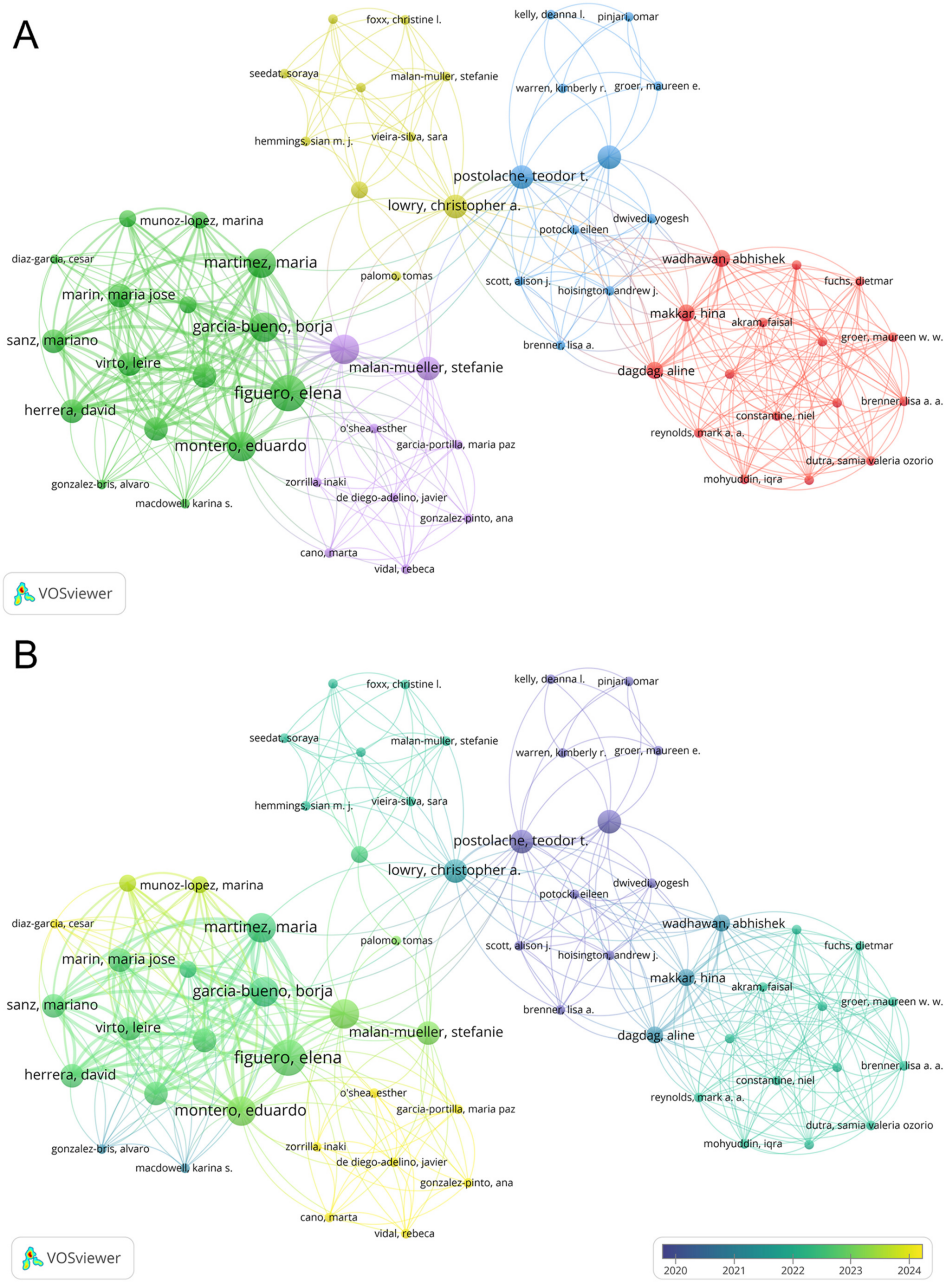
**(A)** The co-authorship network clusters of authors. **(B)** The co-authorship network timeline of authors.

A total of 1,241 researchers have participated in this field of study, among whom 62 demonstrated significant collaboration. We diagrammed the collaboration network of these 62 authors by clustering techniques (Figure 5A). Additionally, an overlay visualization based on the average year of publication was constructed (Figure 5B). As shown in Supplementary Table S1, this study analyzed the top 10 most prolific authors and the most co-cited authors. Ranked by the number of publications, Elena Figuero led with five articles, followed by Borja García-Bueno, Juan C. Leza, María Martínez, and Eduardo Montero, each with four articles in this field. However, among these top 10 authors, Rainer Haak has the highest total and average citation counts. With respect to co-cited authorships, Robert J. Genco, Torbjørn Breivik, and Paul I. Eke ranked in the top three (Supplementary Table S1).

### Analysis of journals

**Figure 6:**
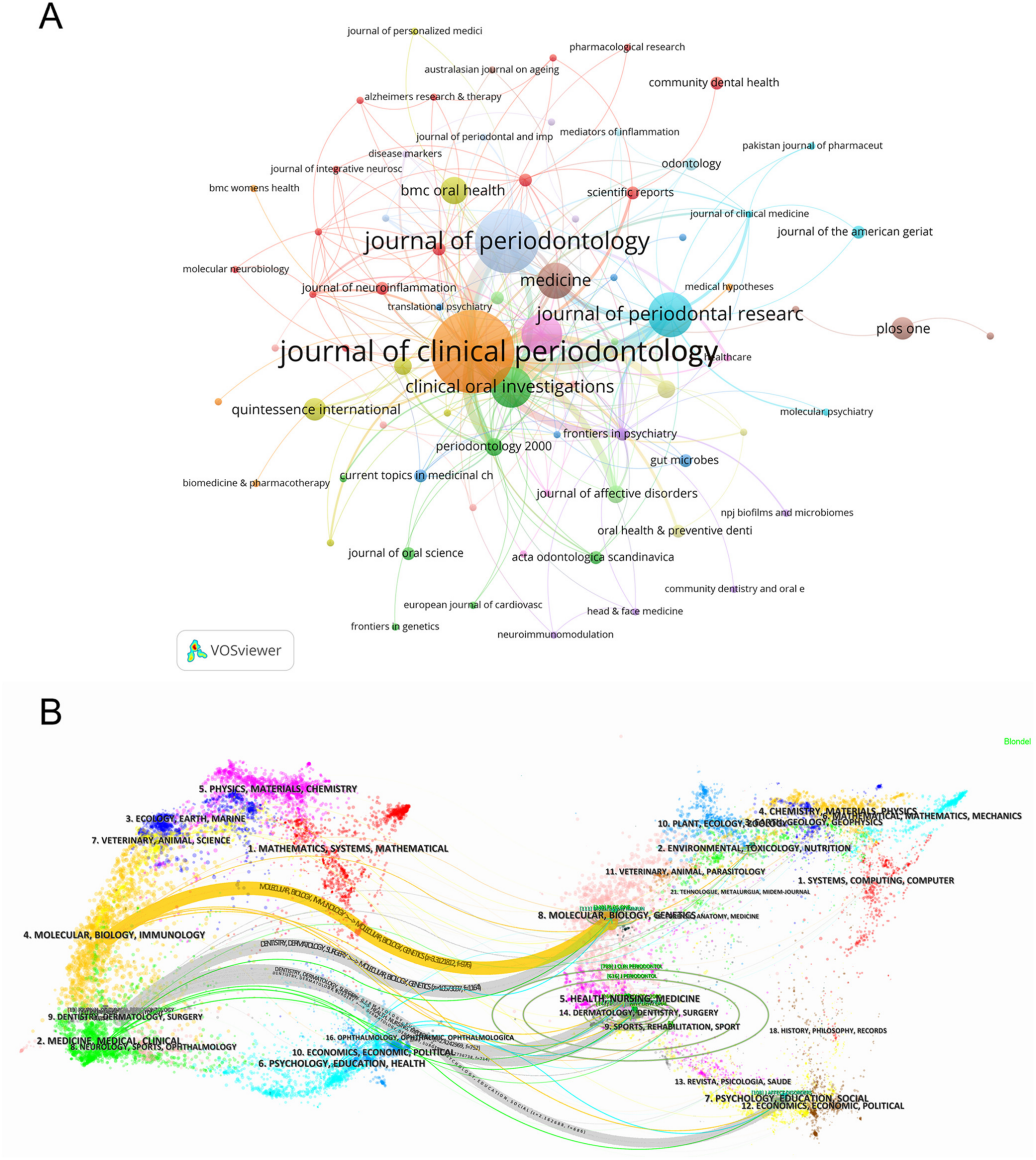
**(A)** The citation map of journals by VOSviewer. **(B)** Double image overlay of journals. The citing journals are located on the left, and the cited journals are located on the right. Different color clusters represent different fields. The link between two clusters means the collaboration between different research fields.

In the field of periodontitis and depression, VOSviewer identified a total of 111 journals. We filtered and visualized the journals, constructing a collaboration network (Figure 6A), where 75 journals exhibited close citation relationships. Table 3 lists the top 10 journals ranked by publication volume and co-citation frequency. The top three journals are the *Journal of Clinical Periodontology*, *Journal of Periodontology*, and the *Journal of Periodontal Research*. The most popular journal, the *Journal of Clinical Periodontology*, and the *Journal of Periodontology* published 19 and 14 articles, respectively, with 610 and 363 citations. Notably, the *Journal of Clinical Periodontology* was the most-cited journal, while Periodontology 2000 attracted more attention in the biosensing field, boasting the highest impact factor.

**Table 3:** Top 10 prolific journals and co-cited journals on the application of periodontitis and depression.

Rank^a^	Journal	Publications	Total citations	Average citations	IF (2023)	Co-cited journal	Co-citations	IF (2023)
1	Journal of Clinical Periodontology	19	610	32.1	5.8	Journal of Clinical Periodontology	789	5.8
2	Journal of Periodontology	14	363	25.9	4.2	Journal of Periodontology	619	4.2
3	Journal of Periodontal Research	9	236	26.2	3.4	Periodontol 2000	266	17.5
4	Clinical Oral Investigations	8	216	27.0	3.1	Journal of Dental Research	216	3.6
5	International Journal of Environmental Research and Public Health	8	38	4.8	NA	Journal of Periodontal Research	179	15.8
6	Medicine	7	133	19.0	1.3	Community Dentistry and Oral Epidemiology	147	8.2
7	Bmc Oral Health	5	9	1.8	2.6	PLoS One	140	2.9
8	Quintessence International	4	37	9.3	1.3	Brain Behavior and Immunity	111	27.4
9	International Dental Journal	3	37	12.3	3.2	Journal of Affective Disorders	108	3.8
10	International Journal of Dental Hygiene	3	30	10.0	1.6	Lancet	91	4.3

^a^ In cases where the number of publications is identical, ranking is determined by the total citations. IF, impact factor.

Using CiteSpace, a dual-map overlay of the journals was generated (Figure 6B). This visualization method bifurcates the citation pathways, with citing journals on the left and cited journals on the right, while simultaneously delineating the thematic focus of the journals. Notably, terms such as “Molecular Biology and Immunology,” “Medicine, Medical, and Clinical,” and “Dentistry, Dermatology, Surgery” from cited journals evolved into “Molecular Biology and Genetics” and “Health, Nursing, Medicine.”

### Analysis of keywords

**Figure 7:**
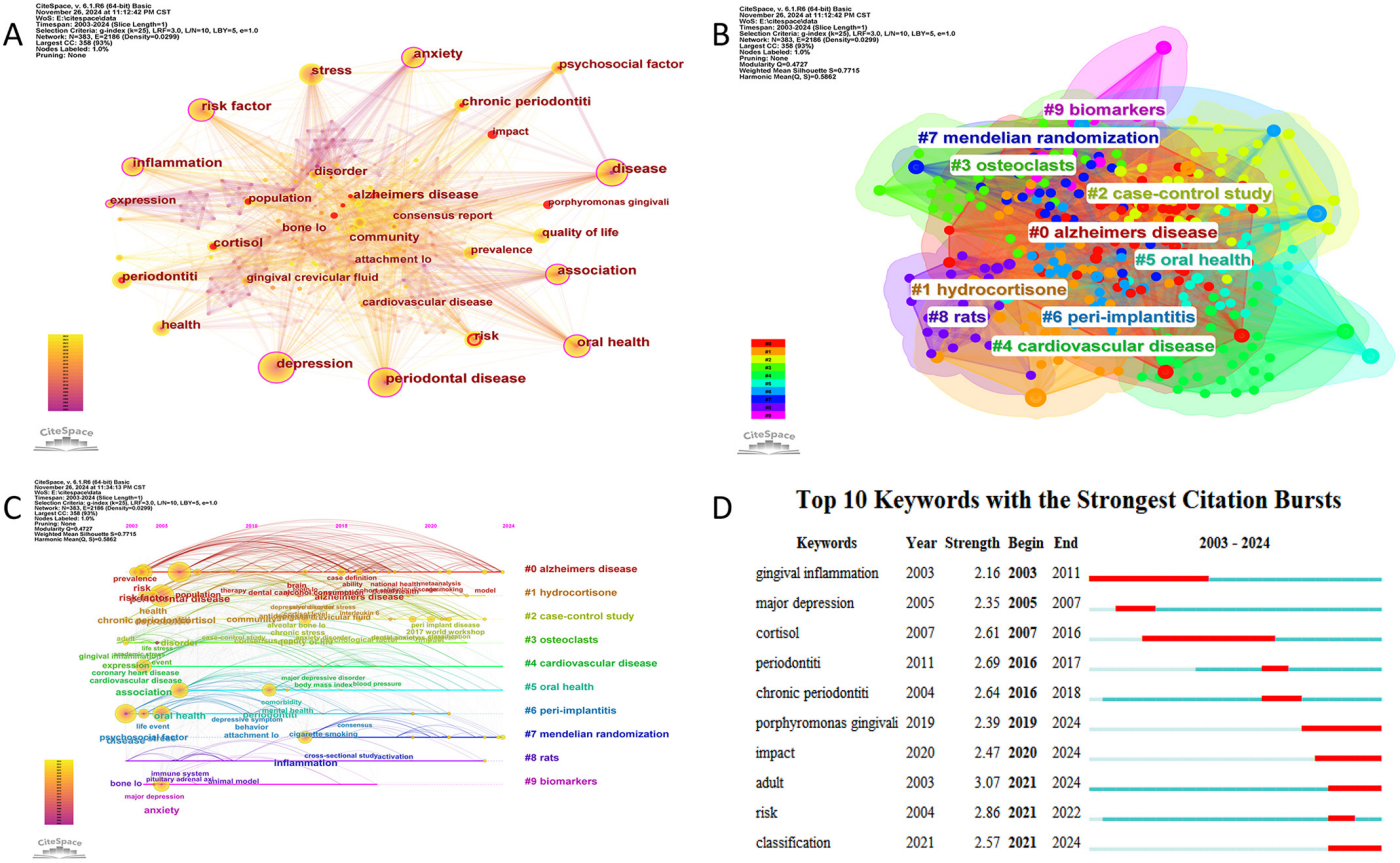
**(A)** The network visualization diagram of keyword co-occurrence analysis. **(B)** Keyword cluster analysis. **(C)** Timeline visualization of references in periodontitis and depression from 2003 to 2024. **(D)** Top 10 keywords with citation bursts.

Using CiteSpace, a co-occurrence network of keywords was generated (Figure 7A), reflecting the relationships among research themes. Nodes with a purple outer ring indicate high centrality, while red nodes represent keywords with citation bursts. The most frequently occurring keywords include “depression” (*n* = 71), “disease” (*n* = 66), “association” (*n* = 50), “oral health” (*n* = 47), and “stress” (*n* = 37). Table 4 lists the top 10 most frequent keywords.

**Table 4:** Top 10 keywords regarding periodontitis and depression.

Keywords	Count of occurrence	Centrality	Year
Depression	71	0.12	2005
Disease	66	0.18	2003
Association	50	0.14	2004
Oral health	47	0.11	2006
Stress	37	0.09	2005
Periodontal disease	36	0.14	2006
Anxiety	34	0.14	2005
Risk factor	31	0.11	2004
Periodontitis	29	0.08	2011
Risk	28	0.06	2004

Keyword clustering analysis can reveal the structural organization and research hotspots within a thematic domain. In this study, we employed the Log-Likelihood Ratio (LLR) clustering method in CiteSpace to perform keyword cluster analysis. The results demonstrated robust clustering validity, with a Modularity Q value of 0.4727 (>0.3) and a Weighted Mean Silhouette score of 0.7715 (>0.7), indicating statistically significant and reliable cluster structures (23). As shown in Figure 7B, a total of 10 distinct clusters were identified, which were #0 alzheimers disease, #1 hydrocortisone, #2 case-control study, #3 osteoclasts, #4 cardiovasclar disease, #5 oral health, #6 peri-implantitis, #7 mendelian randomization, #8 rats, #9 biomarkers.

To further investigate the temporal evolution of research themes, we visualized the dynamic progression of keyword hotspots using a timeline mapping approach (Figure 7C). From 2003 onward, keywords such as “risk factors,” “chronic periodontitis,” and “psychosocial factors” emerged as focal points, highlighting growing scholarly interest in the relationship between periodontitis and psychological influences. Over time, keywords including “depression,” “anxiety,” “oral health,” and “therapeutic efficacy” gained prominence. Notably, after 2013, terms such as “inflammation,” “Alzheimer's disease,” and “smoking” became prevalent, reflecting intensified exploration of potential linkages among periodontitis, systemic disorders, and mental health.

Figure 7D displays the top 10 keywords with the highest burst intensity and their time distribution between 2003 and 2024. Early keywords with citation bursts include “gingival inflammation,” “major depression,” and “cortisol,” reflecting an initial focus on inflammation and psychological factors. Recent citation bursts, such as “impact,” “risk,” and “classification,” indicate a shift toward in-depth exploration and refined analysis of the relationship between periodontitis and depression.

### Analysis of citing publications and references

Supplementary Table S2 summarizes the top 10 most-cited publications, of which six are reviews and only four are original research papers. Notably, a 2016 review by Jianan Hu and his team, published in *Nutrients*, received the highest number of citations. This influential review, titled “*Effect of Probiotics on Depression: A Systematic Review and Meta-Analysis of Randomized Controlled Trials,”* has been widely cited. Additionally, the second-ranked publication by YuChao Chang, published in 2017, titled “*Association between chronic periodontitis and the risk of Alzheimer's disease: a retrospective, population-based, matched-cohort study,”* remains a notable work.

**Figure 8:**
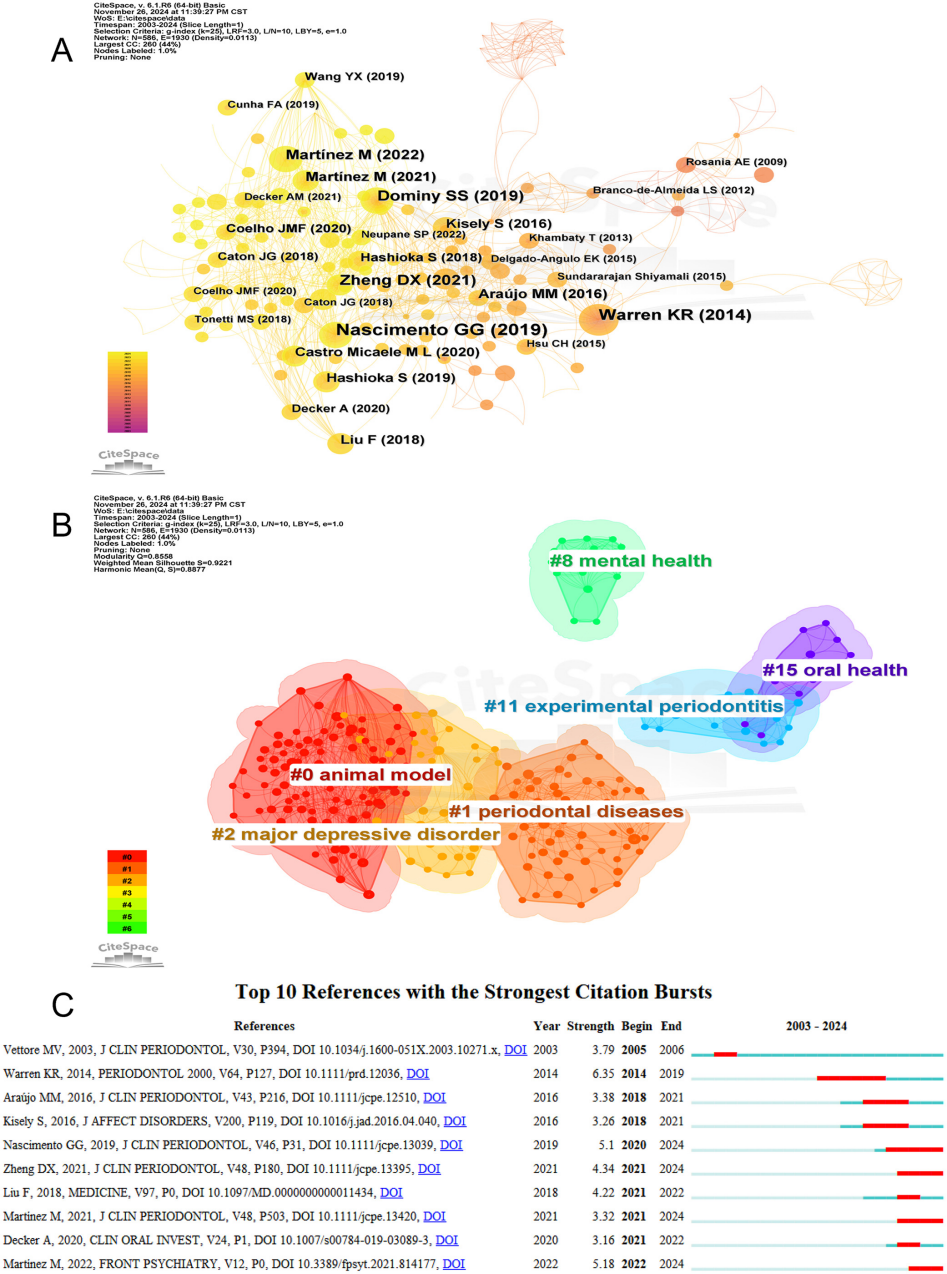
**(A)** The network visualization diagram of co-cited references analysis. **(B)** References cluster analysis. **(C)** Top 10 references with citation bursts.

Using CiteSpace, we plotted a co-citation network of references (Figure 8A), revealing closely related citation relationships between works such as Nascimento GG (2019) and Warren KR (2014). Subsequently, we performed a cluster analysis of the references, categorizing them into several research themes. Figure 8B presents six main clusters: “animal model,” “periodontal diseases,” “major depressive disorder,” “mental health,” and “oral health,” highlighting the progression from animal studies to an in-depth exploration of the relationship between periodontal diseases and depression. A burst-detection analysis identified papers experiencing significant citation surges, pivotal for tracing the evolution of research domains. Figure 8C summarizes the top 10 references with the highest burst intensity between 2003 and 2024. Blue bars indicate the timeline, while red bars denote the periods when articles were widely cited. The earliest citation burst occurred in 2005. Between 2014 and 2019, the article “*Role of chronic stress and depression in periodontal diseases”* experienced the highest burst intensity of 6.35.

## Discussion

This bibliometric analysis provides a comprehensive overview of global research trends concerning the link between periodontitis and depression from 2000 to 2024. The results indicate a substantial increase in scholarly interest over the past decade, with a sharp rise in publications since 2016 and a publication peak in 2024. This growing trend highlights the increasing recognition of the potential bidirectional relationship between oral health and mental health, a domain once underexplored in traditional biomedical research.

China, the USA, and Brazil emerged as the top three most productive countries, collectively accounting for a significant portion of global output. China leads in total publications, while Brazil demonstrated the highest average citations per article, suggesting impactful contributions. The USA's central role in international collaboration reinforces its influence in shaping global research agendas in this field. However, the relatively limited involvement of developing countries suggests geographic disparities in research investment and infrastructure, potentially limiting diverse perspectives in this interdisciplinary domain (11). Institutional analysis revealed that the Universidade de São Paulo and the Institute of Health Carlos III were among the most prolific organizations, with notable contributions from Chinese institutions such as China Medical University and Shanghai Jiao Tong University. Notably, institutions with strong collaboration networks tended to yield higher citation metrics, underscoring the importance of academic partnerships in fostering research visibility and impact. At the author level, while prolific authors such as Elena Figuero and Borja García-Bueno contributed multiple papers, the most influential scholars in terms of citation were identified through co-citation analysis, with Robert J. Genco and Torbjørn Breivik standing out. These findings reflect the interdisciplinary nature of this field, where researchers from dentistry, psychiatry, and immunology converge.

The most frequently cited journals, including the *Journal of Clinical Periodontology* and the *Journal of Periodontology*, suggest that leading periodontal journals serve as major platforms for publishing work related to this topic. The dual-map overlay and keyword evolution analysis further support a shift in research themes—from early explorations of gingival inflammation and psychological factors to more recent focuses on systemic inflammation, Alzheimer's disease, and mechanistic pathways such as cortisol dysregulation and immune response. Keyword co-occurrence and burst analysis provided valuable insight into evolving research hotspots. Clusters such as “Alzheimer's disease,” “mendelian randomization,” and “biomarkers” indicate an expanding focus beyond traditional clinical associations toward mechanistic and genetic explorations. This evolution parallels broader scientific interest in the microbiota-gut-brain axis and its relevance to chronic inflammation and neurodegeneration.

### The mechanistic association between periodontitis and depression

The bidirectional relationship between periodontitis and depression is increasingly supported by mechanistic evidence involving inflammation, neuroimmune interactions, and dysregulation of neuroendocrine signaling pathways.

Studies have suggested that periodontitis may be a potential risk factor for depression (24–27). For individuals with periodontitis, chronic periodontal inflammation can significantly impair daily life, affecting eating, sleep, and overall quality of life, thereby causing substantial distress. Moreover, with the emergence of the COVID-19 pandemic, the associated decline in economic conditions and living standards has led to prolonged psychological stress, further elevating the risk of depression among periodontitis patients (28, 29). In addition, depressive symptoms in individuals with periodontitis are closely associated with disease progression (30).

*Porphyromonas gingivalis* (*Pg*), a major periodontal pathogen (31), has been implicated in a variety of chronic inflammation-based systemic diseases (32). *Pg*-derived lipopolysaccharide (*Pg*-LPS) is a relatively weak agonist of toll-like receptors (TLR) 2 and TLR4, and a moderate activator of nuclear factor kappa-B/signal transducer and activator of transcription (NF-*κ*B/STAT) signaling pathways (33). These characteristics may enable *Pg* to evade innate host defense mechanisms without inducing endotoxin tolerance, thereby contributing to sustained systemic inflammation. Studies have shown that depression in patients with periodontitis is associated with neuroinflammation driven by systemic inflammatory responses. Compared to healthy individuals or periodontitis patients without depression, those with both chronic periodontitis and depression exhibit significantly elevated levels of LPS in the root canal (34), which can trigger the production of various pro-inflammatory cytokines—such as interleukin-1β (IL-1β), interleukin-6 (IL-6), and tumor necrosis factor-α (TNF-α). These cytokines may enter systemic circulation and contribute to the exacerbation of depressive symptoms (35, 36).

Periodontal pathogens and their inflammatory by products can disrupt the integrity of the blood-brain barrier (BBB), leading to brain tissue damage and neuroinflammation (26). The BBB is a specialized protective barrier composed primarily of tightly connected endothelial cells, serving as the brain's first line of defense by preventing pathogens, immune cells, and other harmful substances from entering the central nervous system, thereby maintaining cerebral homeostasis (37). Periodontitis may trigger systemic inflammation through hematogenous dissemination of periodontal bacteria or the translocation of inflammatory mediators from periodontal tissues into the bloodstream (38). Studies have shown that gingipains produced by *Pg* can evade and impair host immune defenses, invade host cells, and release abundant toxic products that disrupt metabolic homeostasis. In the mice model of *Pg*-induced periodontitis, downregulation of albumin D site-binding protein in microglia was found to ameliorate hippocampal inflammation and microglial polarization induced by chronic periodontitis, ultimately alleviating associated behavioral abnormalities, synaptic loss, and impaired neurogenesis in the hippocampus (39). Notably, *Pg* can directly damage vascular endothelial cells, increasing BBB permeability (40). This compromised barrier facilitates the entry of pathogens, peripheral nerves, and immune cells into the brain from leaky regions lacking BBB integrity, thereby impairing the maturation of brain-derived neurotrophic factors (BDNF) and heightening the risk of neuroinflammation (41).

BDNF, a key member of the neurotrophin family, plays a critical role in neuronal network formation and synaptic plasticity. A reduction in neurotrophic factors impairs the brain's ability to adapt to environmental stimuli, thereby contributing to the onset of depression. Compared to healthy individuals, patients with depression typically exhibit lower serum levels of BDNF, which significantly increase following treatment with antidepressants (42) (43),. Pg has been shown to induce depressive-like behaviors by inhibiting BDNF maturation through downregulation of the P75 neurotrophin receptor in astrocytes (44). Furthermore, inflammatory cytokines can suppress BDNF expression (45), exerting adverse neuromodulatory effects (46). These findings highlight the complex interplay between inflammatory processes, neurotrophic signaling, and depression, suggesting that both Pg and proinflammatory cytokines may contribute to the pathophysiology of depression by impairing BDNF signaling and neuronal plasticity.

In addition, patients with depression often exhibit dysfunction of the hypothalamic-pituitary-adrenal (HPA) axis. Hyperactivation of the HPA axis leads to elevated cortisol levels (47, 48), which can delay periodontal wound healing, suppress immune-inflammatory responses, promote alveolar bone resorption, and reduce the regenerative capacity of periodontal tissues, thereby accelerating the progression of periodontitis (30, 49). A recent study further demonstrated that under stress conditions, upregulation of norepinephrine and Pg virulence factors synergistically increases IL-1β production by microglial cells, potentially explaining how psychological stress may exacerbate the progression of periodontitis-associated systemic inflammatory diseases (14).

Collectively, these findings suggest that periodontal pathogens and their inflammatory mediators contribute to a vicious cycle of systemic and neuroinflammation, HPA axis dysregulation, and neurotrophic impairment. This multifaceted interaction underscores the need for integrated therapeutic approaches targeting both oral and mental health domains.

### The clinical relevance of the association between periodontitis and depression

Currently, an increasing number of researchers are focusing on the correlation between these two diseases from the perspective of clinical management. Periodontitis is a multifactorial infectious disease primarily caused by dental plaque biofilms. LPS derived from periodontal pathogens can stimulate host immune responses and induce neuroinflammation. Therefore, regular non-surgical periodontal therapy aimed at controlling dental plaque remains an effective strategy for managing periodontitis.

For patients with depression, greater awareness of oral hygiene should be encouraged, along with efforts to reduce smoking and recognize the increased risk of periodontitis due to compromised immune function. Adoption of healthy lifestyle habits—such as smoking and alcohol cessation, a balanced diet, and targeted health education—may help maintain oral microbiome homeostasis and reduce the risk of periodontal disease.

Given the shared involvement of inflammation in both periodontitis and depression, and the known anti-inflammatory effects of certain antidepressants (50), increasing attention has been directed toward the use of antidepressant therapy in patients suffering from both conditions. The therapeutic effects of antidepressants in depression are largely attributed to their ability to modulate the breakdown and reuptake of various neurotransmitters; however, their immunomodulatory potential has also garnered interest in periodontal research.

Among these agents, the tricyclic antidepressant tianeptine has been shown to alleviate periodontal inflammation in olfactory bulbectomy-induced depressive rats. Tianeptine significantly reduced bacterial LPS-induced expression of the pro-inflammatory cytokine TNF-α, while increasing anti-inflammatory cytokine levels, thereby attenuating the severity of periodontitis and improving depression-related behaviors (17).

Similarly, oral administration of desipramine in rats with experimentally induced periodontitis modulated the local immune response in periodontal tissues. Desipramine downregulated the expression of inflammatory mediators such as IL-1β, inducible nitric oxide synthase (iNOS), and cyclooxygenase-2 (COX-2), and also reduced dendritic cell antigen-presenting capacity and T lymphocyte proliferation, resulting in decreased periodontal tissue destruction (16). Furthermore, a cross-sectional clinical study demonstrated that fluoxetine treatment in humans led to reduced expression of IL-1β, matrix metalloprotein-9, and NF-κB transcriptional activity in periodontal tissues, suggesting a notable anti-inflammatory effect (15).

These findings collectively indicate that pharmacological interventions aimed at treating depression may offer additional benefits in the management of periodontitis. Such dual-effect strategies provide a promising therapeutic approach for patients suffering from both depression and periodontal disease.

In patients with periodontitis, reducing local and systemic inflammation through basic periodontal therapy—such as scaling and root planing—may help alleviate depressive symptoms. Combining periodontal treatment with psychological interventions, such as cognitive behavioral therapy, can synergistically improve both depressive symptoms and oral health outcomes through multidimensional mechanisms (51). Taken together, these insights support a novel, interdisciplinary treatment model for patients suffering from both periodontitis and depression, underscoring the importance of collaborative care between dental and mental health professionals.

## Future perspectives and conclusion

In mechanistic studies on the association between depression and periodontitis, researchers have proposed the concept of the “brain–oral axis,” suggesting that the anatomical proximity between the oral cavity and the brain may facilitate the influence of oral microbiota on the brain, potentially triggering neurological disorders. The Oral-Gut-Brain axis can be conceptualized as a dynamic network of anatomical and physiological interactions linking the oral cavity, gastrointestinal tract, and central nervous system. The oral-brain axis primarily involves the trigeminal nerve, while the gut-brain axis is predominantly mediated by the vagus nerve. Communication between the oral cavity and the gut is largely anatomical in nature and facilitated by the continuous ingestion of oral microbiota. Collectively, these systems form a bidirectional communication network in which the oral-brain and gut-brain pathways are interconnected and mutually influential (52).

Moreover, emerging evidence suggests that periodontitis can induce alterations in gut microbiota composition, modulate the intestinal immune system, and impair gut barrier function. These changes may increase intestinal permeability, trigger mucosal immune responses, and facilitate the translocation of virulence factors or pro-inflammatory mediators—such as IL-1β, IL-6, interleukin-6 (IL-17), and TNF-α—into the peripheral circulation (53), eventually reaching the brain and initiating neuroinflammation. High-throughput 16S rRNA gene sequencing has revealed that patients with periodontitis exhibit reduced gut microbial diversity and compositional shifts compared to healthy controls, notably characterized by the enrichment of Verrucomicrobia and Proteobacteria and an increased Firmicutes/Bacteroidetes ratio (54). These findings indicate that periodontitis-induced gut dysbiosis can compromise intestinal barrier integrity, stimulate systemic and neuroinflammatory responses, and potentially contribute to the pathophysiology of depression. However, the underlying mechanisms and therapeutic implications of this relationship remain to be fully elucidated. Research on the oral–gut–brain axis is anticipated to become a key focus in this field moving forward.

Although the precise nature of the relationship between periodontitis and depression remains under investigation, a growing body of evidence suggests a significant association between the two conditions. Importantly, association does not necessarily imply causation, and further research is needed to establish a definitive causal link. Nonetheless, current bibliometric analyses offer valuable insights into recent trends, enhancing our understanding of the periodontitis–depression relationship and identifying potential directions for future research. This deeper understanding may provide new perspectives for clinical practice, enabling dental professionals to recognize the potential link between oral health and depression. These efforts could help lay the groundwork for integrated treatment strategies targeting both oral and mental health, ultimately improving patient outcomes.

Despite substantial progress in this field, several limitations remain. First, among the most frequently cited publications, review articles dominate, indicating a lack of high-quality interventional or longitudinal studies to clarify causal relationships. Second, although the number of publications has grown rapidly, the translation of research findings into clinical practice remains limited. Third, research resources are concentrated in a few countries and institutions, which may result in publication bias and highlight the need to enhance the participation of developing countries and underrepresented populations in related research.

In summary, research on the relationship between periodontitis and depression is currently in a phase of rapid expansion, characterized by sustained growth in research output, increasing interdisciplinary integration, and diversified research themes. Future work should focus on mechanistic validation, well-designed clinical trials, and international collaboration to promote the translation of basic research into clinical practice, ultimately aiming to improve both oral and mental health in a synergistic manner.

## Data Availability

The original contributions presented in the study are included in the article/Supplementary Material, further inquiries can be directed to the corresponding authors.
