# Supplementary material for: Interdisciplinary research on periodontitis and depression: a bibliometric analysis of research trends, hotspots and future directions
Source: Front Oral Health. 2025 Jun 27;6:1588737. doi: 10.3389/froh.2025.1588737 (PMC12245893; doi:10.3389/froh.2025.1588737)
Supplement: Supplementary file 1 [file Table1.docx]

**Table S1**. Top 10 prolific authors and co-cited authors on the application of periodontitis and depression.

| **Rank ^a^** | **Author** | **Publications** | **Total Citations** | **Average Citations** | **Country** | **Co-Cited Author** | **Co-Citations** | **Country** |
| --- | --- | --- | --- | --- | --- | --- | --- | --- |
| 1 | [Elena Figuero](https://pubmed.ncbi.nlm.nih.gov/?sort=date&size=50&term=Figuero+E&cauthor_id=39436969) | 5 | 85 | 17.0 | Spain | [Robert J Genco](https://pubmed.ncbi.nlm.nih.gov/?sort=date&size=50&term=Genco+RJ&cauthor_id=29539798) | 81 | USA |
| 2 | [Borja García-Bueno](https://pubmed.ncbi.nlm.nih.gov/?sort=date&size=50&term=Garc%C3%ADa-Bueno+B&cauthor_id=39436969) | 4 | 85 | 21.3 | Spain | [Torbjørn Breivik](https://pubmed.ncbi.nlm.nih.gov/?sort=date&size=50&term=Breivik+T&cauthor_id=25713634) | 45 | Norway |
| 3 | [María Martínez](https://pubmed.ncbi.nlm.nih.gov/?sort=date&size=50&term=Mart%C3%ADnez+M&cauthor_id=39436969) | 4 | 85 | 21.3 | Spain | [Paul I Eke](https://pubmed.ncbi.nlm.nih.gov/?sort=date&size=50&term=Eke+PI&cauthor_id=35900929) | 44 | USA |
| 4 | [Juan C Leza](https://pubmed.ncbi.nlm.nih.gov/?sort=date&size=50&term=Leza+JC&cauthor_id=39368974) | 4 | 73 | 18.3 | Spain | [Michael Maes](https://pubmed.ncbi.nlm.nih.gov/?sort=date&size=50&term=Maes+M&cauthor_id=39557130) | 41 | China |
| 5 | [Eduardo Montero](https://pubmed.ncbi.nlm.nih.gov/?sort=date&size=50&term=Montero+E&cauthor_id=39436969) | 4 | 41 | 10.3 | Spain | [Steve Kisely](https://pubmed.ncbi.nlm.nih.gov/?sort=date&size=50&term=Kisely+S&cauthor_id=39359160) | 38 | Australia |
| 6 | Sadayuki Hashioka | 3 | 124 | 41.3 | Japan | [Annsofi Johannsen](https://pubmed.ncbi.nlm.nih.gov/?sort=date&size=50&term=Johannsen+A&cauthor_id=16937592) | 36 | Sweden |
| 7 | [Omer Cakmak](https://pubmed.ncbi.nlm.nih.gov/?sort=date&size=50&term=Cakmak+O&cauthor_id=30187981) | 3 | 48 | 16.0 | Turkey | [Maurizio S Tonetti](https://pubmed.ncbi.nlm.nih.gov/?sort=date&size=50&term=Tonetti+MS&cauthor_id=38987924) | 33 | Italy |
| 8 | [Rainer Haak](https://pubmed.ncbi.nlm.nih.gov/?sort=date&size=50&term=Haak+R&cauthor_id=38002804) | 3 | 45 | 15.0 | Germany | [Marconi Gomes da-Silva](https://pubmed.ncbi.nlm.nih.gov/?sort=date&size=50&term=da-Silva+MG&cauthor_id=36793470) | 31 | Brazil |
| 9 | [Nagore Ambrosio](https://pubmed.ncbi.nlm.nih.gov/?sort=date&size=50&term=Ambrosio+N&cauthor_id=39436969) | 3 | 41 | 13.7 | Spain | [George Hajishengallis](https://pubmed.ncbi.nlm.nih.gov/?sort=date&size=50&term=Hajishengallis+G&cauthor_id=38838669) | 30 | USA |
| 10 | [David Herrera](https://pubmed.ncbi.nlm.nih.gov/?sort=date&size=50&term=Herrera+D&cauthor_id=39436969) | 3 | 41 | 13.7 | Spain | [Ana Cristina de Oliveira Solis](https://pubmed.ncbi.nlm.nih.gov/?sort=date&size=50&term=Solis+ACO&cauthor_id=26616397) | 30 | Brazil |

^a^ In cases where the number of publications is identical, ranking is determined by the total citations

**Table S2**. Top 10 most cited papers on the application of periodontitis and depression research.

| **Rank^a^** | **Title** | **Type** | **Total Citations** | **Journal** | **IF (2023)** | **Corresponding Author** | **Affiliation** | **Year** |
| --- | --- | --- | --- | --- | --- | --- | --- | --- |
| 1 | Effect of Probiotics on Depression: A Systematic Review and Meta-Analysis of Randomized Controlled Trials | Review | 312 | [Nutrients](https://www.letpub.com.cn/index.php?page=journalapp&view=detail&journalid=8652) | 4.8 | [Jianan Hu](https://pubmed.ncbi.nlm.nih.gov/?term="Hu J"[Author]) | Central South University | 2016 |
| 2 | Association between chronic periodontitis and the risk of alzheimer's disease: a retrospective, population-based, matched-cohort study | Article | 185 | [Alzheimers Research & Therapy](https://www.letpub.com.cn/index.php?page=journalapp&view=detail&journalid=8737) | 7.9 | [Yuchao Chang](https://pubmed.ncbi.nlm.nih.gov/?sort=date&size=50&term=Chang+YC&cauthor_id=28784164) | Chung Shan Medical University | 2017 |
| 3 | Modifiable risk factors in periodontitis: at the intersection of aging and disease | Review | 127 | Periodontol 2000 | 17.5 | [Mark A Reynolds](https://pubmed.ncbi.nlm.nih.gov/?sort=date&size=50&term=Reynolds+MA&cauthor_id=23040337) | UCLA School of Dentistry | 2014 |
| 4 | No mental health without oral health | Review | 119 | [Canadian Journal of Psychiatry-Revue Canadienne de Psychiatrie](https://www.letpub.com.cn/index.php?page=journalapp&view=detail&journalid=1482) | 3.3 | [Steve Kisely](https://pubmed.ncbi.nlm.nih.gov/?sort=date&size=50&term=Kisely+S&cauthor_id=27254802) | University of Queensland | 2016 |
| 5 | Using probiotics in clinical practice: Where are we now? A review of existing meta-analyses | Review | 116 | Gut Microbes | 12.2 | [Mariangela Rondanelli](https://pubmed.ncbi.nlm.nih.gov/?sort=date&size=50&term=Rondanelli+M&cauthor_id=28640662) | University of Pavia | 2017 |
| 6 | Is depression associated with oral health outcomes in adults and elders? A systematic review and meta-analysis | Article | 96 | Clinical Oral Investigations | 3.1 | [Mariana Gonzalez Cademartori](https://pubmed.ncbi.nlm.nih.gov/?sort=date&size=50&term=Cademartori+MG&cauthor_id=30191327) | Federal University of Pelotas | 2018 |
| 7 | Prevalence of peri-implantitis in medically compromised patients and smokers: a systematic review | Review | 91 | International journal of oral & maxillofacial implants | 1.7 | [Alberto Turri](https://pubmed.ncbi.nlm.nih.gov/?sort=date&size=50&term=Turri+A&cauthor_id=26800167) | Göteborg University | 2016 |
| 8 | Role of chronic stress and depression in periodontal diseases | Review | 85 | Periodontol 2000 | 17.5 | [Kimberly R Warren](https://pubmed.ncbi.nlm.nih.gov/?sort=date&size=50&term=Warren+KR&cauthor_id=22595870) | University of Maryland School of Medicine | 2014 |
| 9 | Porphyromonas gingivalis and Its Systemic Impact: Current Status | Article | 81 | Porphyromonas gingivalis | 3.3 | [Feng Mei](https://pubmed.ncbi.nlm.nih.gov/?sort=date&size=50&term=Mei+F&cauthor_id=33202751) | Huazhong University of Science and Technology | 2020 |
| 10 | Tooth loss and dementia: a critical examination | Article | 73 | Journal of dental research | 5.7 | [W M Thomson](https://pubmed.ncbi.nlm.nih.gov/?sort=date&size=50&term=Thomson+WM&cauthor_id=11014902) | The University of Otago | 2021 |

^a^ The ranking is determined by the total citations.
